# Self-Medication and Contributing Factors Among Pregnant Women Attending Antenatal Care at Public Hospitals of Harar Town, Ethiopia

**DOI:** 10.3389/fphar.2018.01063

**Published:** 2018-09-25

**Authors:** Abera Jambo, Getnet Mengistu, Mekonnen Sisay, Firehiwot Amare, Dumessa Edessa

**Affiliations:** ^1^Department of Clinical Pharmacy, School of Pharmacy, College of Health and Medical Sciences, Haramaya University, Harar, Ethiopia; ^2^Department of Pharmacy, College of Medicine and Health Sciences, Wollo University, Dessie, Ethiopia; ^3^Department of Pharmacology and Toxicology, School of Pharmacy, College of Health and Medical Sciences, Haramaya University, Harar, Ethiopia

**Keywords:** self-medication, conventional medicine, herbal medicine, pregnant women, contributing factors

## Abstract

**Background:** Self-medication has been increasing in many developing and developed countries. Its use during pregnancy presents a major challenge due to potential undesirable effects on mother and the fetus. So the aim of this study was to assess the prevalence of self-medication and contributing factors, among pregnant women.

**Methodology:** Institution based cross sectional study was conducted among 244 pregnant women attending antenatal care at Hiwot Fana Specialized University Hospital and Jugal Hospital from February to March, 2017. A structured questionnaire based interview was used to collect data from each study subject. Then, data were categorized and analyzed using SPSS version 20 software. Logistic regression analysis was used to determine the significance of the association between the outcome and independent variables. *P*-value <0.05 was considered as a statistically significant in multivariate analysis.

**Results:** The prevalence of self-medication during current pregnancy was 69.4%; out of which, 40.6% uses only herbal medicines to self-medicate. Time saving (50.7%) and prior experience of the drug (25.35%) were the main reasons for self-medication using conventional medicines while fewer side effects (59.86%) and effectiveness (35.92%) were the common reasons for self-medication using herbal medicines. Common cold and headache were among the common indications for self-medication. Friends (28.17%) and the pharmacist/druggist (23.94%) were the commonest source of information for conventional medicines while family/friends (69.72%) and neighbors (26.76%) were the common source of information for herbal medicines. Community drug retail outlets and neighbors were the commonly used sources of conventional medicines; while market place and self-preparation were the common sources of herbal medicines. Previous history of self-medication was significantly associated (*P* < 0.05) with current self-medication with conventional drugs and being a farmer by occupation and poor monthly income were significantly associated with herbal medicine use during pregnancy (*P* < 0.05).

**Conclusion:** The prevalence of self-medication during pregnancy was very high in this study which showed a need for public trainings for all women of reproductive age about the risks of inappropriate self-medication.

## Introduction

Worldwide, self-medication is practiced by different population groups. Pregnant women are among those exposed to self-medication and medication intake presents a challenge and a concern due to altered drug pharmacokinetics and drugs crossing the placenta, possibly causing harm to the fetus (Bánhidy et al., 2005). Self-medication is the selection and use of medicines (conventional and/or traditional) by individuals to treat self-recognized illnesses or symptoms (World Health Organiztion [WHO], 2018). Self-medication is a universal challenge that requires attention because of the potential threat, not only to the woman but also to her unborn child. In most developing countries where the health system is not well-organized, the likelihood that women will self-medicate is high; as many drugs are contraindicated in pregnancy and women may not know which drugs are dangerous to them and their unborn child (Emmanuel et al., 2014).

In addition to conventional medicines, pregnant women also use herbal medicines which are the main category of traditional medicine. Herbal medicines are plant derived preparations with therapeutic or other human health benefits; and contain raw or processed ingredients from one or more sources (World Health Organiztion [WHO], 2000). A WHO survey revealed that about 70–80% of the world’s population rely on non-conventional medicines mainly of herbal sources in their primary healthcare (Chan et al., 2003). Pregnant women are among those populations exposed to herbal medicines to manage pregnancy related ailments. In Africa, including Ethiopia, more pregnant women use herbal medicines to treat pregnancy related problems due to their cost-effectiveness and easy access. In total, 80% of the Ethiopian population use traditional medicine and around 90% of all deliveries are managed by traditional birth attendants or relatives. Nevertheless, data available for the use of herbal medicines among pregnant women in Ethiopia are scarce (Bayisa et al., 2014).

Assessment of drug use during pregnancy is important for clinical, educational, economic, and public health purposes. The aim of this study was to assess the prevalence of self-medication and contributing factors among pregnant women. The results of this study will help health care providers in educating and counseling pregnant women about the consequence of self-medication. In particular, this study will help to establish baseline information that may be useful to midwives and other health workers in educating pregnant women. It also provides baseline data about the current prevalence of self-medication among pregnant women and related factors.

## Materials and Methods

### Study Setting and Period

The study was conducted at HFSUH and JH, Harar town, Eastern Ethiopia, which is found at 526 km from Addis Ababa (capital city of Ethiopia). While there are different wards and clinics within the hospitals, the study was conducted in the antenatal clinic (ANC) of both hospitals from February 20 to March 20, 2017.

### Study Design

An institution based cross sectional study was conducted using a structured questionnaire based interview of pregnant women who were attending the ANC at HFSUH and JH.

### Population

All pregnant women who attend ANC at the two hospitals were the potential source and study populations.

### Selection Criteria

Pregnant women willing to participate in the study were included in the study but mentally ill pregnant women were excluded from the study.

### Sample Size Determination and Sampling Technique

The sample size was determined using a single proportion formula and a prevalence of self-medication; 73.1% (*P* = 0.73) (Laelago et al., 2016) with a margin of error of 5% (*d* = 0.05) and a 95% confidence interval. Since the total number of pregnant women attending the hospitals’ ANC were <10,000 (approximately 1,061), by applying a reduction formula and adding a 5% contingency (for non-response rate), the final sample size was determined to be 247. Simple random sampling was used to select the study participants from the study population.

### Study Variables

Prevalence of self-medication was the dependent variable and socio-demographic characteristics (age, marital status, ethnicity, religion, occupation, monthly income, place of residence, and distance from health facilities), obstetric characteristics (stage of pregnancy, gravidity, number of children, and history of abortion) and previous history of self-medication were the independent variables.

### Data Collection Instrument and Procedure

Data were collected using semi-structured interviewer administered questionnaire. The questionnaire was prepared in English language including all relevant variables based on the study objectives and then translated to local languages (Afaan Oromo and Amharic language). To check for loss or change of meaning back-translation was made and there was no loss or change of meaning. Questionnaires, pen and pencil were used during data collection.

### Data Quality Control

After the questionnaire was prepared, a pretest pilot was performed for validity and reliability, with modification undertaken accordingly. After data collection, the data were cleaned and checked for completeness and consistency before data processing and analysis.

### Data Processing and Analysis

After collection was completed, data were categorized and analyzed by using SPSS version 20 software. Descriptive statistics including frequencies, percentages, mean and standard deviations (SD) were used to summarize study variables and evaluate distribution of responses. Logistic regression was used to analyze the association between independent variables and prevalence of self-medication using crude odds ratio (COR) and adjusted odds ratio (AOR) at 95% confidence level. A *P*-value of <0.05 was considered statistically significant. Data were then presented using tables and figures.

## Results

### Socio-Demographic Characteristics of the Respondents

A total of 247 pregnant women were invited in to the study, with a response rate of 98.8%. Out of 244 pregnant women who participated in the study, 41.4% were in the 24–29 year age group, with a median age of 25 years; and most (91%) were married. With regard to their occupation, 43% were private workers and 84% had a low monthly income according to the WHO income scale level. In addition, 95 (38.9%) attended primary education (**Table 1**).

**Table 1:** Socio-demographic characteristics of pregnant women attending ANC at HFSUH and JH, from February 20 to March 20, 2017 (*n* = 244).

Variables		*N*	%	
Age (years)	18–23	83	34.0	Median age (±SD) 25 ± 4.68
	24–29	101	41.4	
	30–35	47	19.3	
	>35	13	5.3	
Marital status	Married	222	91.0	
	Divorced	22	9.0	
Occupational status	Private	105	43.0	
	Government employee	25	10.2	
	House wife	71	29.1	
	Farmer	35	14.3	
	Student	8	3.3	
Monthly income (ETB)^∗^	<1,380 (poor)	33	13.5	Mean income (±SD) 2,513.77 ± 1,388.46
	1,381–6,900 (low)	205	84.0	
	6,901–13,800 (middle)	6	2.5	
Educational status	Illiterate	40	16.4	
	Primary (1–8)	95	38.9	
	Secondary (9–12)	78	32.0	
	College/university students	7	2.9	
	Diploma/degree	24	9.8	
Place of residence	Urban	197	80.7	
	Rural	47	19.3	
Distance from health facility	<5 km	171	70.1	
	5–10 km	67	27.5	
	>10 km	6	2.5	


*^∗^Classification is according to WHO income level scale for developing countries. ETB, Ethiopian birr.*

### Obstetric Information of the Respondents

Among the 244 pregnant women interviewed, 84 (34.4%) were gravida two and 39 (16%) were gravida four and above. From a total of 174 pregnant women with gravida two and above, 82 (47.1%) had one child and 26 (14.9%) had a history of previous abortion, of which 15 (57.7%) were due to unknown illnesses (**Table 2**).

**Table 2:** Obstetric characteristics of pregnant women attending ANC at HFSUH and JH, from February 20 to March 20, 2017.

Variables	Frequency (*N*)	Percentage (%)
**Gravidity (*n* = 244)**		
One	70	28.7
Two	84	34.4
Three	51	20.9
≥Four	39	16.0
**Parity (*n* = 174)**		
No child	11	6.3
One child	82	47.1
Two children	48	27.6
More than two children	33	19.0
**History of abortion (*n* = 174)**		
Yes	26	14.9
No	148	85.1
**Reason for abortion (*n* = 26)**		
Unknown illness	15	57.7
Unwanted pregnancy	10	38.5
Use of unspecified medication	1	3.8
**Stage of pregnancy (*n* = 244)**		
1st trimester	47	19.3
2nd trimester	106	43.4
3rd trimester	91	37.3


### Self-Medication Practice

#### Prevalence of Self-Medication

From a total of 244 pregnant women interviewed, 170 (69.7%) practice self-medication during current pregnancy and among 174 pregnant women with gravida two and above, 133 (76.4%) practice self-medication during previous pregnancy. Herbal medicines were used most commonly in both cases (**Table 3**).

**Table 3:** Prevalence of self-medication among pregnant women attending ANC at HFSUH and JH, February 20–March 20, 2017.

Self-medication	Frequency (*N*)	Percentage (%)
**During current pregnancy (*n* = 244)**	170	69.7
Conventional medicines	28	11.5
Herbal medicines	99	40.6
Both	43	17.6
**During previous pregnancy (*n* = 174)**	133	76.4
Conventional medicines	23	13.2
Herbal medicines	71	40.8
Both	39	22.4


#### Self-Medication With Conventional Medicines

Among the 244 pregnant women interviewed, 71 (29.1%) practiced self-medication using conventional medicines during current pregnancy while from a total of 174 pregnant women with gravida two and above, 62 (35.6%) practiced during previous pregnancy. The most common reason to self-medicate were to save time (50.7%), prior experience with the drug (25.4%), better knowledge about the disease and treatment (21.1%), and easy drug availability (2.8%).

**FIGURE 1:**
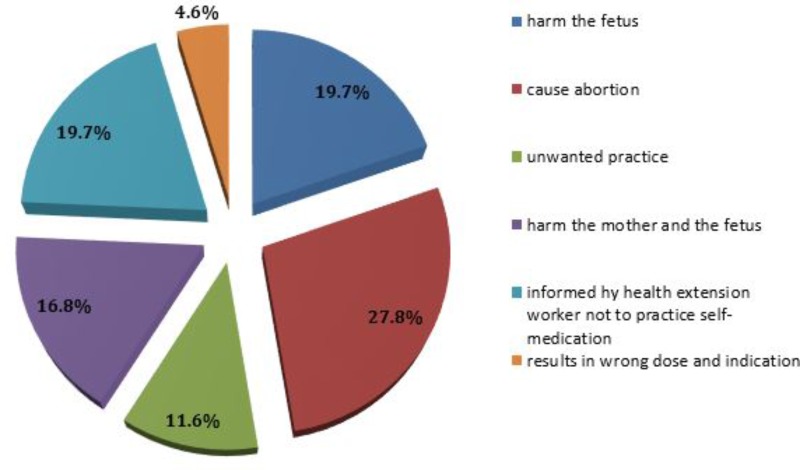
Reasons for not practicing self-medication during pregnancy among pregnant women attending ANC at HFSUH and JH, February 20–March 20, 2017 (*n* = 173).

From a total of 173 pregnant women who did not practice self-medication during their current pregnancy, 48 (27.8%) believed that self-medication with conventional medicines during pregnancy causes abortion and 8 (4.62%) believed that it results in a wrong dose and indication (**Figure 1**).

**FIGURE 2:**
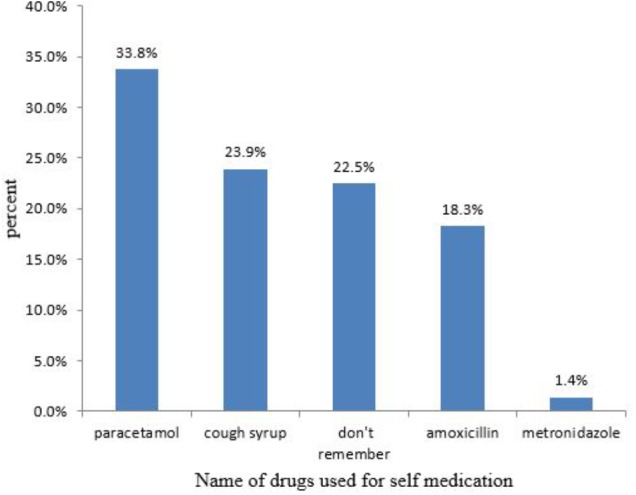
Drugs used for self-medication among pregnant women attending ANC at HFSUH and JH, February 20–March 20, 2017 (*n* = 71).

Among the 71 pregnant women who practiced self-medication during their current pregnancy, 42.3% used the drug to treat a common cold and 36.6% treated a headache (**Table 4**), with the most widely used drugs; paracetamol (33.8%) followed by cough syrup (23.9%) (**Figure 2**).

**Table 4:** Indication for self-medication using conventional medicines among pregnant women attending ANC at HFSUH and JH, February 20–March 20, 2017 (*n* = 71).

Indication for self-medication	Frequency (N)	Percent (%)
Common cold	30	42.3
Headache	26	36.6
Nausea/vomiting	10	14.1
Other^∗^	5	7.0


*^∗^UTI, typhoid fever, diarrhea, and amebiasis.*

**FIGURE 3:**
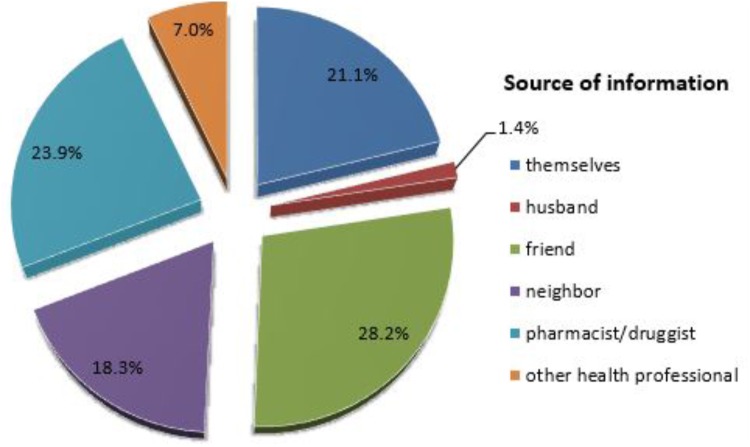
Source of information for self-medication among pregnant women attending ANC at HFSUH and JH, February 20–March 20, 2017 (*n* = 71).

Among pregnant women who practice self-medication with conventional medicines during their current pregnancy, the most common source of information were their friends (28.2%) (**Figure 3**) and 71.8% got the drugs from CDROs (**Table 5**).

**Table 5:** Sources of drugs for self-medication among pregnant women attending ANC at HFSUH and JH, February 20–March 20, 2017 (*n* = 71).

Sources of drugs	Frequency	Percent
CDROs	51	71.8
Neighbors	10	14.1
Friends’	4	5.6
Shops	6	8.5


*CDROs, community drug retail outlets.*

Among these women, 32 (45.1%) knew the recommended dose and 15 (21.1%) knew side effects of the drug, while 24 (33.8%) had no information about the drug they had used.

In this study, multivariate logistic regression analysis shows that previous history of self-medication was significantly associated with current self-medication. Women with a previous history of self-medication were 65.6 fold more likely to practice self-medication than those with no previous history of self-medication [AOR: 65.64, 95% CI (19.071–225.93)] (**Table 6**).

**Table 6:** Factors associated with self-medication practice during pregnancy among pregnant women attending ANC at HFSUH and JH, February 20–March 20, 2017.

Variables		Self-medication		COR (95% CI)	AOR (95% CI)
				
		No (%)	Yes (%)		
Marital status	Married	163 (94.2)	59 (83.1)	1.00	1.00
	Divorced	10 (5.8)	12 (16.9)	3.315 (1.361–8.077)^∗^	0.525 (0.107–2.570)
Occupation	Private	66 (38.2)	39 (54.9)	1.00	1.00
	House wife	54 (31.2)	17 (23.9)	0.533 (0.272–1.045)	0.443 (0.122–1.600)
	Farmer	28 (16.2)	7 (9.9)	0.423 (0.169–1.059)	0.482 (0.106–2.191)
	Governmental	20 (11.6)	5 (7.0)	0.423 (0.147–1.217)	0.643 (0.091–4.518)
	Student	5 (2.9)	3 (4.2)	1.015 (0.230–4.483)	0.752 (0.07–15.399)
Gravida	Gravida I	50 (28.9)	20 (28.2)	0.759 (0.382–1.507)	–
	Gravida II	55 (31.8)	29 (40.8)	1.00	1.00
	Gravida III	35 (20.2)	16 (22.5)	0.867 (0.412–1.823)	0.337 (0.090–1.265)
	Gravida IV and above	33 (19.0)	6 (8.5)	0.345 (0.130–0.918)^∗^	0.062 (0.12–0.308)
Stage of pregnancy	1st trimester	28 (16.2)	19 (26.8)	1.719 (0.837–3.531)	0.948 (0.286–3.145)
	2nd trimester	76 (43.9)	30 (42.3)	1.00	1.00
	3rd trimester	69 (39.6)	22 (31)	0.808 (0.426–1.531)	0.542 (0.164–1.792)
Previous self-medication	No	103 (83.7)	9 (17.6)	1.00	1.00
	Yes	20 (16.3)	42 (82.4)	24.033 (10.123–57.061)^∗^	65.641 (19.071–225.93)^∗^


*^∗^*P* < 0.05 statistically significant.*

#### Self-Medication With Herbal Medicines

Among 244 pregnant women interviewed, 142 (58.2%) used herbal medicines to self-medicate during their current pregnancy; and from a total of 174 pregnant women with two and above gravida, 110 (63.2%) had a history of herbal medicine use during a previous pregnancy. Among 102 pregnant women who don’t use herbal medicines during the current pregnancy, 40.2% believed that self-medication with herbal medicines may cause harm to the fetus and only one pregnant woman believed that herbal medicines causes harm to both the mother and her fetus (**Table 7**).

**Table 7:** Reason for not using herbal medicines among pregnant women attending ANC at HFSUH and JH, February 20–March 20, 2017 (*n* = 102).

Reason for not using herbal medicines	Frequency	Percent
Unwanted practice	23	22.5
May cause harm to the fetus	41	40.2
Have no information about herbal medicine use	17	16.7
Difficult to know dose of herbal medicine	13	12.7
cause abortion	7	6.9
Cause harm to both mother and her fetus	1	1.0


**FIGURE 4:**
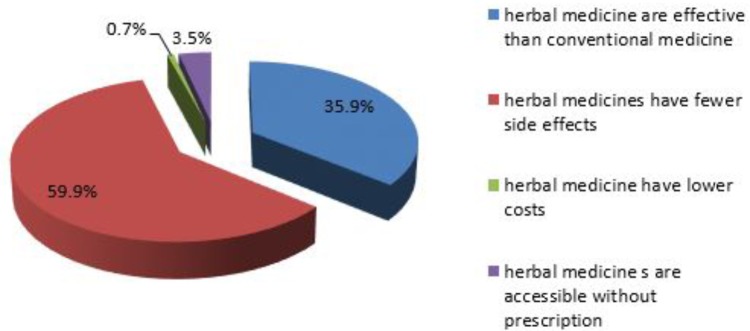
Reason for using herbal medicine among pregnant women attending ANC at HFSUH and JH, February 20–March 20, 2017 (*n* = 142).

Of 142 pregnant women who used herbal medicines to self-medicate during their current pregnancy, 59.9% believed that herbal medicines have fewer side effects and 35.9% believed that herbal medicines are more effective than conventional medicine (**Figure 4**). Among these women, 63.4% used the herbs to treat the common cold followed by headache (44.4%), with the most widely used herb; ginger (62%) followed by tena’adam (53.5%) (**Table 8**).

**Table 8:** Indications for herbal medicine and name of herbs used among pregnant women attending ANC at HFSUH and JH, February 20–March 20, 2017 (*n* = 142).

Herbal medicines		Frequency	Percent
Indication^a^	Common cold	90	63.4
	Headache	63	44.4
	Nausea/vomiting	16	9.9
	Other^b^	3	2.1
Name of herbs used^a^	Ginger (*zingiber officinale*)	88	62.0
	Tena’adam (*ruta chalepensis*)	76	53.5
	Garlic (*allium sativa*)	63	44.4
	Damakase (*Ocimum lamiifolium*)	10	7.0
	Tosign (*thymus vulgaris*)	1	0.7


*^*a*^Participant used more than one herb for more than one disease. ^*b*^UTI and to facilitate labor; % > 100.*

The most common sources of information about herbal medicines for these women was their family (69.7%); and the market place was the most common source of herbs (48.6%) (**Table 9**).

**Table 9:** Source information about herbal medicines and source of herbs among pregnant women attending ANC at HFSUH and JH, February 20–March 20, 2017 (*n* = 142).

Variable	Category	Frequency	Percent (%)
Source of information	Family and friends	99	69.7
	Neighbors	38	26.8
	Others^a^	5	3.5
Source of herbs	Market place	69	48.6
	Self-preparation	56	39.4
	Neighbors	17	12


*^*a*^Others: traditional healers and health professionals.*

Multivariate logistic regression analysis revealed that pregnant women who were farmer in occupation were 8.6 times more likely to use herbal medicines when compared to private workers [AOR: 8.634, 95% CI (1.552–48.030)]. The analysis also revealed that pregnant women with poor monthly income (<1,380) were 3.7 times more likely to use herbal medicines when compared to women with low monthly income (1,381–6,900) [AOR: 3.67, 95% CI (1.064–12.678) (**Table 10**).

**Table 10:** Factors associated with herbal medicine use among pregnant women attending ANC at HFSUH and JH, February 20–March 20, 2017 (*n* = 142).

Variables		Herbal medicine use		COR (95% CI)	AOR (95% CI)
				
		No (%)	Yes (%)		
Monthly income	<1,380	10 (9.8)	23 (16.2)	1.696 (0.768–3.745)^∗^	3.673 (1.064–12.678)^∗^
	1,381–6,900	87 (85.3)	118 (83.1)	1.00	1.00
	6,901–13,800	5 (4.9)	1 (0.7)	0.147 (0.017–1.285)	0.000 (0.000)
Occupation	Private	51 (50)	54 (38)	1.00	1.00
	Governmental	12 (11.8)	13 (9.2)	1.023 (0.427–2.449)	1.923 (0.201–18.429)
	House wife	33 (32.4)	38 (26.8)	1.088 (0.595–1.988)	0.650 (0.281–1.507)
	Farmer	3 (2.9)	32 (22.5)	10.074 (2.904–34.94)^∗^	8.634 (1.552–48.030)^∗^
	Student	3 (2.9)	5 (3.5)	1.574 (0.358–6.926)	6.088 (0.329–112.699)
Education level	Illiterate	8 (7.8)	32 (22.5)	2.786 (1.160–6.689)^∗^	1.355 (0.414–4.436)
	Primary	39 (38.2)	56 (39.4)	1.00	1.00
	Secondary	38 (37.3)	40 (28.2)	0.733 (0.401–1.340)	0.849 (0.381–1.894)
	College/university	4 (3.9)	3 (2.1)	0.522 (0.111–2.465)	0.217 (0.006–7.518)
	Diploma/degree	13 (12.7)	11 (7.7)	0.589 (0.239–1.451)	0.988 (0.103–9.456)
Gravidity	Gravida I	37 (36.3)	33 (23.2)	0.637 (0.336–1.207)	–
	Gravida II	35 (34.3)	49 (34.5)	1.00	1.00
	Gravida III	24 (23.5)	27 (19)	0.804 (0.399–1.619)	0.163 (0.026–1.034)
	Gravida IV and above	6 (5.9)	33 (23.2)	3.929 (1.486–10.384)	0.194 (0.012–3.008)
Parity	No child	5 (7.7)	6 (5.5)	0.894 (0.252–3.166)	0.530 (0.128–2.202)
	One child	35 (5.8)	47 (43.1)	1.00	1.00
	Two children	21 (32.3)	27 (24.8)	0.957 (0.467–1.965)	4.323 (0.675–27.668)
	≥Three children	4 (6.2)	29 (26.6)	5.399 (1.738–16.768)	15.934 (0.876–289.856)


*^∗^P < 0.05 statistically significant.*

## Discussion

The prevalence of self-medication among pregnant women during current pregnancy was 69.7%, which is higher than the results of studies done in Ethiopia (Bayisa et al., 2014; Befekadu et al., 2014; Abeje et al., 2015; Laelago et al., 2016) and other countries (Liao et al., 2015; Mbarambara et al., 2016; Joseph et al., 2017). The main reason for this difference is that our study included both conventional and herbal medicines for self-medication but other studies included either of them. Self-medication can cause significant challenges for the individuals and community, especially in women during pregnancy (Ebrahimi et al., 2017) and it is necessary to aware all women of reproductive age about the dangers and side effects of self-medication by providing public trainings.

Based on the types of drug used, self-medication was classified in to two in this study; self-medication with conventional medicines and self-medication with herbal medicines. The reason for this classification is the difference in factors that facilitate the practice of self-medication in both cases. Accordingly, there are different factors which facilitate self-medication with conventional medicines. In this study, time saving and prior experience of the drug were the two most common reasons for self-medication. The result is comparable to studies conducted at Jima Specialized University Hospital (JSUH) (Befekadu et al., 2014), governmental health centers in Bahir Dar city (Abeje et al., 2015) and at selected hospitals in Jos, Nigeria (Emmanuel et al., 2014). Easy access of drugs without prescription and prescribing excessive drugs are among the factors that facilitate self-medication (Ebrahimi et al., 2017).

In this study, the most common ailments for which the pregnant women practiced self-medication were the common cold, headache and nausea/vomiting. This result is in line with studies conducted at JSUH (Befekadu et al., 2014), Bukavu, Eastern DR Congo (Mbarambara et al., 2016) and at selected hospitals in Jos, Nigeria (Emmanuel et al., 2014) where headache, fever, gastrointestinal disorders, infections, and the common cold were among common ailments for which the pregnant women practiced self-medication. Due to physiological disturbances, most pregnant women are affected by cold, cough, fever, nausea and vomiting or any other minor ailments and it is better to consult her doctor before proceeding to take any medications.

These finding shows, paracetamol (33.8%), cough syrup (23.94%), and amoxicillin (18.31%) were among commonly used medications for self-medication during pregnancy. The result is similar with the study done at JSUH (Befekadu et al., 2014) and Bukavu, Eastern DR Congo (Mbarambara et al., 2016), which showed paracetamol and amoxicillin were among the commonly used medications for self-medications during pregnancy. Despite its high prevalence during pregnancy, different researches indicate that prolonged use of paracetamol may cause different problems on the fetus. For instance, long-term maternal use of paracetamol during pregnancy was substantially associated with Attention deficient hyperactive disorder, ADHD (Ystrom et al., 2017) and increased risk of overall cerebral palsy and unilateral spastic cerebral palsy (Liew et al., 2016; Petersen et al., 2017). As a result of such kinds of evidences, self-medication with paracetamol during pregnancy should be with caution and the pregnant women should advised physicians.

Findings of our study revealed that friends, pharmacist/druggist, self-recommendation and neighbors were the common source of information for self-medication which is similar to study conducted at JSUH (Befekadu et al., 2014), which revealed that self-recommendation followed by pharmacist/druggists were the common source of information for self-medication. In this study, CDROs, neighbors, shops and friends were the commonly used source of drugs for self-medication during pregnancy. This finding is consistent with most findings (Befekadu et al., 2014; Abeje et al., 2015; Mbarambara et al., 2016).

The result of this study revealed that there is a significant association between previous history of self-medication and current self-medication (*P* < 0.001) which is consistent with a previous study conducted at JSUH (Befekadu et al., 2014). This might be due to the fact that pregnant women with previous self-medication practice had more experience to self-medicate than that didn’t practice it.

In this study the prevalence of herbal medicine use among pregnant women was 58.2%. This was within the range of 22.3 to 82.3% reported by a review of four articles from the Middle East (John and Shantakumari, 2015) and it was higher than the study in a tertiary hospital in Imo state, South-East, Nigeria (36.8%) (Duru et al., 2016), in family health centers in Alexandria (27.3) (Orief et al., 2014) and in a study conducted at Nekemte Hospital (50.4%) (Bayisa et al., 2014), but lower than study done in Hossana Town (73.1%) (Laelago et al., 2016), in Nigeria (67.5%) (Fakeye et al., 2009) and in herbal clinics in Gucha district, Kenya (68.9%) (Ondicho et al., 2016). The difference might be due to variance in availability of herbs and cultural differences among study participants.

In our study, fear of harms to the fetus, considering it as an undesirable practice and lack of information about herbal medicine use were the most common reasons for not using herbal medicine during pregnancy. This is consistent with a study conducted among pregnant women attending a tertiary hospital in Northern Nigeria (Tamuno et al., 2011). In contrast, our results showed fewer side effects with herbal medicine use and the perceived effectiveness of herbal medicine were the most common reasons for herbal medicine use among pregnant women. The result is in line with studies conducted among pregnant women attending an ANC at Nekemte Hospital, Hossana town and in Nigeria (Fakeye et al., 2009; Bayisa et al., 2014; Laelago et al., 2016) and in line with a review of four articles from the Middle East (John and Shantakumari, 2015). Since the safety of most herbal medicines isn’t well-established (e.g., tena’adam), this perception may expose the fetus to unknown potential risks.

The study showed ginger (*zingiber officinale*), tena’adam (*ruta chalepensis*), and garlic (*allium sativa*) were the common herbs used during pregnancy. The finding is comparable with studies done at Nekemte hospital and Hossana town (Bayisa et al., 2014; Laelago et al., 2016) in Ethiopia and tertiary Hospital in Northern Nigeria (Tamuno et al., 2011). Other studies also indicate ginger as the most extensively studied botanical for nausea and vomiting. A systematic review of six published clinical trials found that ginger, at doses of 1.0 to 1.5 g, is effective for reducing nausea and vomiting. It was also used for cold and flu’s, perhaps due to its diaphoretic properties, health promotion and gastrointestinal disorders (Borrelli et al., 2005). Another multinational study showed that ginger was one of the most frequently used herbal medicine classified as safe (Kennedy et al., 2013) and the results of a large cohort study and clinical evidence in human pregnancy had not found any harmful effect of ginger to mother or fetus (Heitmann et al., 2013; Viljoen et al., 2014). Studies in human pregnancy have also shown no adverse effect of garlic (Mills et al., 2013; Aalami-Harandi et al., 2015). The main problem with the use of herbal medicines are contamination, mis-labeling and there isn’t standardized dose especially in developing country like Ethiopia. For instance, in this study the participants found information from family, friends and neighbors and got the drug from market place or prepare by themselves which indicates that there is no standardization of dose and poor regulatory framework for importation, manufacturing and distribution of herbal medicines within a country. This may expose the mother and the fetus to harmful effects. As our knowledge, the safety and effectiveness of tena’adam isn’t yet established and needs further study to use in pregnant women.

Multivariate logistic regression analysis revealed that pregnant women who were farmer in occupation and pregnant women with poor monthly income were more likely to use herbal medicine when compared to pregnant women with other occupation (*P* = 0.014) and with low monthly income (*P* = 0.04), respectively. This might be due to farmers have more access for self-preparation of herbal medicine and might be due to less affordability of herbal medicine.

### Strength and Limitation of the Study

Unlike previous studies, which targeted only self-medications with conventional medicines, our study includes both conventional and herbal medicines to self-medicate. The limitation of this study might be a small sample size, as a larger sample size would have provided greater insight into less commonly used medications that might have the potential for more serious harm to the mother or fetus. The study didn’t identify the frequency of self-medications. Most of the medication and herbal products are likely safe with limited use, but could cause harm depending on the dose and duration of self-medication.

## Conclusion

The prevalence of self-medication in pregnant women was very high in this study which showed a need for public trainings for all women of reproductive age about the risks of inappropriate self-medication. In addition, health care providers, especially those that are involved in ANC should be aware of evidence regarding potential benefits or harm of herbal medicinal agents when used by pregnant women. This study recommends a detailed study on commonly used herbs to establish the efficacy and safety of these herbs to ensure the well-being of the mother and fetus by concerned bodies.

## Ethics Statement

The ethical clearance and study approval was obtained from Haramaya University, College of Health and Medical Sciences, School of Pharmacy (C/AC/R/D/01/164/17). Permission letters were also received from Harare regional health offices and respective hospital administrators (HFSUH, JH) to conduct this study. Verbal informed consent was obtained from study participants after the objectives of the study were made clear. Names of study participants were omitted from the data collection form to ensure confidentiality.

## Author Contributions

AJ and GM conceived the original idea and drafted the proposal. AJ, GM, MS, FA, and DE were involved in data acquisition, analysis, interpretation, and write up of the paper. GM drafted the manuscript and prepared it for publication. All authors read and approved the final version of the manuscript.

## Conflict of Interest Statement

The authors declare that the research was conducted in the absence of any commercial or financial relationships that could be construed as a potential conflict of interest.

## Abbreviations

CDROs: community drug retail outlets
HFSUH: Hiwot Fana Specialized University Hospital
JH: Jugal Hospital
JUSH: Jimma University Specialized Hospital
WHO: World Health Organization
